# High-Dose Chemotherapy Followed by Peripheral and/or Bone Marrow Stem Cell Transplant in Patients With Advanced Sarcoma: Experience of a Canadian Centre

**DOI:** 10.1080/13577140410001710521

**Published:** 2004

**Authors:** Sébastien J. Hotte, Anne M. Smith, Vivien H.C. Bramwell, Kang Howson-Jan

**Affiliations:** 1 Department of Medicine McMaster University and Division of Medical Oncology Hamilton Regional Cancer Centre (HRCC) 699 Concession Street Hamilton Ontario L8V 5C2 Canada; 2 Department of Oncology Queen's University and Division of Medical Oncology Kingston Regional Cancer Centre (KRCC) Kingston Ontario Canada; 3 Department of Medicine University of Western Ontario Division of Medical Oncology LRCC and Division of Hematology, London Health Science Centre London Ontario Canada

## Abstract

*Purpose*: Few reports have been published on the evaluation of stem cell auto transplantation for chemosensitive sarcomas. Some suggest benefit, others do not. We present results of 24 patients with sarcoma undergoing autotransplantation at a Canadian institution.

*Patients and Methods*: Twenty-four patients were treated between 1988 and 1998: 23 were ≥18 years (median 27; range 12–56); genders were equal; 12 patients had Ewing's sarcoma. At diagnosis, 12 (50%) had metastatic disease. Prior to autotransplant, all had ≥1 chemotherapy regimen. Fourteen (58%) were in complete remission (CR) and seven (29%) had minimal residual disease. All received etoposide 60 mg/kg (Day –4), melphalan 140 mg/mα^2^ on (Day –3) and a stem cell reinfusion (Day 0).

*Results*: Three patients (12.5%) were alive and disease-free with median follow-up of 92 months (80–142); one was alive with disease 32 months post-autotransplant. Twenty had died (disease, 17; transplant-related, 2; unknown, 1). Of the four alive, three had Ewing's sarcoma, one alveolar rhabdomyosarcoma, and all were in CR at transplant. Median time to relapse was 6 months (2–59). Sixteen of 18 (89%) relapsed within 1 year. Median overall survival was 10 months (0–137). A trend towards improved survival (*P*=0.07) was evident for patients in CR prior to autotransplant.

*Conclusions*: Stem cell autotransplantation does not benefit most patients with sarcoma. A subgroup of high-risk patients in CR may fare better and warrant further study.


*Sarcoma*, June/September 2004, *VOL. 8, NO. 2/3, 63–69*

Taylor & Francis healthsciences

ORIGINAL ARTICLE

### High-dose chemotherapy followed by peripheral and/or bone marrow stem cell transplant in patients with advanced sarcoma: experience of a Canadian Centre

SÉBASTIEN J. HOTTE[1], ANNE M. SMITH[2], VIVIEN H.C. BRAMWELL[1] & KANG HOWSON-JAN[3]

[1]*Department of Medicine, McMaster University and Division of Medical Oncology, Hamilton Regional Cancer Centre (HRCC), Hamilton, Ontario, Canada;* [2]*Department of Oncology, Queen's University and Division of Medical Oncology, Kingston Regional Cancer Centre (KRCC), Kingston, Ontario, Canada;* [3]*Department of Medicine, University of Western Ontario, Division of Medical Oncology, LRCC and Division of Hematology, London Health Science Centre, London, Ontario, Canada*

**Abstract**

*Purpose*: Few reports have been published on the evaluation of stem cell auto transplantation for chemosensitive sarcomas. Some suggest benefit, others do not. We present results of 24 patients with sarcoma undergoing autotransplantation at a Canadian institution.

*Patients and Methods*: Twenty-four patients were treated between 1988 and 1998: 23 were $\geq$18 years (median 27; range 12–56); genders were equal; 12 patients had Ewing's sarcoma. At diagnosis, 12 (50%) had metastatic disease. Prior to autotransplant, all had $\geq$1 chemotherapy regimen. Fourteen (58%) were in complete remission (CR) and seven (29%) had minimal residual disease. All received etoposide 60 mg/kg (Day −4), melphalan 140 mg/m$^2$ on (Day −3) and a stem cell reinfusion (Day 0).

*Results*: Three patients (12.5%) were alive and disease-free with median follow-up of 92 months (80–142); one was alive with disease 32 months post-autotransplant. Twenty had died (disease, 17; transplant-related, 2; unknown, 1). Of the four alive, three had Ewing's sarcoma, one alveolar rhabdomyosarcoma, and all were in CR at transplant. Median time to relapse was 6 months (2–59). Sixteen of 18 (89%) relapsed within 1 year. Median overall survival was 10 months (0–137). A trend towards improved survival ($P = 0.07$) was evident for patients in CR prior to autotransplant.

*Conclusions*: Stem cell autotransplantation does not benefit most patients with sarcoma. A subgroup of high-risk patients in CR may fare better and warrant further study.

**Key words:** *adults, sarcoma, high-dose chemotherapy, stem cell transplant*

### Introduction

Sarcomas are rare tumors, and their incidence shows a bimodal distribution. They occur in children and young adults (0–20 years), but the highest numerical incidence is between ages 50 and 80 years. Six thousand new soft tissue sarcomas, 250 Ewing's sarcomas, 750 osteosarcomas and 450 chondrosarcomas are diagnosed yearly in the U.S.[1]

The initial management of patients with sarcomas usually includes surgery. Radiotherapy and/or chemotherapy may also be added before or after surgery. Although sarcomas have historically been described as relatively chemoresistant, those seen predominantly in the pediatric population, e.g., embryonal rhabdomyosarcomas, osteosarcomas and Ewing's sarcomas, can be responsive to chemotherapy. Despite best efforts, inoperable locally advanced and metastatic sarcomas are rarely curable with conventional therapy. For adult soft tissue sarcomas, the median survival from recognition of metastatic disease can be quite variable but tends to be in the order of 8–12 months in most series[2–6]. However, a small but significant proportion will experience longer survival and up to a quarter of patients will be alive 2 years following a diagnosis of

Correspondence to: Sébastien J. Hotte, MD, FRCPC, Division of Medical Oncology, Hamilton Regional Cancer Centre, 699 Concession Street, Hamilton, Ontario, Canada L8V 5C2. Tel.: +1-905-387-9495, ext. 64602; Fax: +1-905-575-6326; E-mail: sebastien.hotte@hrcc.on.ca

1357-714X print/1369–1643 online © 2004 Taylor & Francis Ltd
DOI: 10.1080/13577140410001710521

metastatic disease. In common with many other tumors, some factors tend to predict a worse prognosis with regards to response to treatment and survival of patients with advanced soft tissue sarcomas (ASTS). These include a non-pediatric histology, non-extremity primary sites, poor response to chemotherapy, liver metastases and poor performance status.[7]

Multiple chemotherapeutic agents have been studied in the treatment of ASTS.[2–7] Unfortunately, most of these agents have shown only marginal response rates. In order to achieve better response rates and increase survival, higher dose chemotherapy regimens have been evaluated. There has been some evidence of a dose–response relationship with anthracyclines, first from a report by the MD Anderson Cancer Centre[8] and then reproduced in randomized trials by other centres.[9,10] However, this has not been universally observed: a study of the EORTC Soft Tissue and Bone Sarcoma Group failed to show a dose–response relationship with epirubicin.[11] Similar data have also been seen for ifosfamide.[12–16] With regards to melphalan, a clear dose effect was shown in rhabdomyosarcoma xenograft models,[17] and in non-randomized series of pediatric tumors a response rate of 30% has been achieved with higher doses.[18] Unfortunately, no survival benefits have been seen.

The suggestion of a possible dose–response relationship has driven many centers across the world to study and implement very high dose chemotherapy regimens followed by bone marrow transplantation. Only a few series have been published to date.[19–32] Most of these were retrospective studies accruing very small patient numbers, and no prospective randomized controlled trials have been published. Results of these series are conflicting, some clearly concluding that high dose chemotherapy and bone marrow transplant offers no advantage, others suggesting that survival benefits may exist, although only by comparison with historical controls. These positive trials have tended to include a high proportion of pediatric-type sarcomas as well as a variable percentage of patients with no evaluable disease at the time of transplant, this modality being used as consolidation therapy. Another confounding issue has been the variety of conditioning regimens used. Some series describe the use of a single chemotherapeutic regimen for all their patients receiving high-dose chemotherapy while others were not as restrictive and used a variety of regimens within the same series. The most popular chemotherapeutic agent is melphalan, most often combined with etoposide. Total-body irradiation is sometimes used in conjunction with high-dose chemotherapy.

Given the uncertainties of the previously published series as well as a desperate need to improve disease-free survival in patients with advanced sarcomas, the London Regional Cancer Centre (LRCC) selectively offered high-dose chemotherapy (HDCT) followed by autologous stem cell transplant (ASCT) in the late 1980s and 1990s. We herein present the results of this retrospective, observational series.

### Patients and methods

### *Eligibility*

Patients should be in complete remission (CR) or have minimal residual disease at time of transplant. These patients should also be at high risk for relapse or metastatic disease and must have also shown chemosensitivity with previous treatments.

### *Patient characteristics*

To identify subjects, the charts of all patients having received an autologous stem cell transplant at the LRCC were reviewed, and patients with a diagnosis of sarcoma were identified. Patient characteristics at time of diagnosis are summarized in Table 1. Twenty-four patients with advanced sarcoma received myeloablative chemotherapy and ASCT between 1988 and 1998. Twelve were female and 12 were male with a median age of 27 years (range, 12–56 years). At diagnosis, 12 patients (50%) had Ewing's sarcoma of bone. The rest had a variety of histological entities, which included three patients with primitive neuroectodermal tumors (PNET), alveolar and embryonal rhabdomyosarcomas (three patients each), as well as one patient each with leiomyosarcoma, spindle cell sarcoma and neurosarcoma. Eleven patients (46%) had metastatic disease at diagnosis, while 11 others (46%) had locally advanced disease, e.g., bulky disease without lymph node involvement. Of the two remaining patients,

**Table 1.** *Patient characteristics at time of diagnosis*

| Variable ($N = 24$) | Value (range or percentage) |
|---|---|
| **Parameters at diagnosis:** | |
| *Median age (years)* | 24 (range, 11–53) |
| *Histological diagnosis:* | |
| Ewing's sarcoma | 12 (50%) |
| PNET | 3 (12.5%) |
| Alveolar rhabdomyosarcoma | 3 (12.5%) |
| Embryonal rhabdomyosarcoma | 3 (12.5%) |
| Leiomysarcoma | 1 (4.2%) |
| Spindle cell sarcoma | 1 (4.2%) |
| Neurosarcoma | 1 (4.2%) |
| *Disease involvement:* | |
| *Metastases:* | 12 (50%) |
| Lungs only | 8 (33%) |
| Bones only | 1 (4.2%) |
| Lungs and bones | 1 (4.2%) |
| Lymph nodes only | 1 (4.2%) |
| Lymph nodes and bones | 1 (4.2%) |
| *Locally advanced disease* | 11 (46%) |
| *Localized disease* | 2 (8.3%) |

one received high-dose chemotherapy and ASCT because of disease relapse with subsequent good but incomplete response to chemotherapy while the other had a high-risk histological diagnosis (leiomyosarcoma) initially treated with surgery, with metastatic recurrence in the lungs treated by metastatectomy after a good partial response to etoposide. Informed consent was obtained from all patients.

*Prior therapy*

All 24 patients were previously treated with chemotherapy prior to referral for stem cell transplant. Most patients had been heavily pretreated prior to referral with a median number of cycles received of nine (range, 2–18 cycles). All had either anthracyclines, ifosfamide, or etoposide-based therapy. Twenty patients received the VAC regimen (vincristine, adriamycin, cyclophosphamide), 18 patients received ifosfamide and etoposide was used in 17 patients. Eight patients (33%) received two or more regimens. A total of 42% of the patients received prior radiation therapy to the site of their primary disease.

*Status at bone marrow transplant*

At time of ASCT, the median age was 27 years with all but one patient being at least 18 years old. Other patient characteristics at time of ASCT are seen in Table 2. The median interval between diagnosis and ASCT was 15 months (range, 9–120 months).

At time of BMT, 15 of the 24 patients (62.5%) of patients had no evaluable disease. Most were at high risk of relapse because of a locally advanced or metastatic presentation and received HDCT and BMT as consolidative therapy. One patient had localized disease at first presentation but had the high-risk histological diagnosis of alveolar rhabdomyosarcoma. Seven patients (29%) had residual disease but had had a good response to chemotherapy. Two patients did not meet the planned eligibility criteria as they had progressive disease but were accepted for HDCT and ASCT as a last resort.

**Table 2.** *Patient characteristics at time of transplant*

| Variable ($N = 24$) | Value (range or percentage) |
|---|---|
| **Parameters at ASCT:** | |
| *Median age (years)* | 27 (range, 12–56) |
| *Median interval since diagnosis (months)* | 15 (range, 9–120) |
| *Disease status:* | |
| No evaluable disease | 15 (62.5%) |
| Residual disease with PR to chemo | 7 (29%) |
| Progressive disease | 2 (8%) |
| *Previous therapy:* | |
| ≥1 regimen of chemotherapy | 24 (100%) |
| 2 or more regimens | 8 (33%) |
| Median number of cycles | 9 (range, 2–18) |
| Radiation therapy | 10 (42%) |
| *Types of chemotherapy regimen:* | |
| VAC | 20 (83%) |
| Ifosfamide | 18 (75%) |
| Etoposide | 17 (71%) |

PR, partial response; VAC, vincristine, adriamycin, cyclophosphamide.

*High-dose chemotherapy regimen*

Patients had bone marrow and/or peripheral blood stem cell (PBSC) collection prior to high-dose chemotherapy. The first 19 patients only had bone marrow stem cell collection alone. The last five patients were transplanted in 1998 and all had attempts at PBSC collection following 5 days of G-CSF mobilization. Target PBSC yield was $2.5 \times 10^6$ CD34+ cells/kg. Three of the five patients had insufficient CD34-positive cells in their PBSC collection alone and had combined PBSC/autologous bone marrow (ABM) reinfusion. One patient had an adequate PBSC collection and one patient failed to mobilize CD34-positive cells with G-CSF and received ABMT only. Patients requiring ABMT had bone marrow harvesting performed under general anaesthesia 2–4 weeks before ASCT. The bone marrow was cryopreserved in DMSO (dimethyl sulfoxide) and stored in liquid nitrogen at −196°C. The stem cells were thawed in 40°C water bath at the bedside. Reinfusion occurred over a maximum of 20 min. Bag-injected DNAse was used to disaggregate WBC clumps prior to reinfusion. Prior to bone marrow harvest, bone marrow biopsies were performed for all patients and were negative for malignancy.

All 24 patients received the same myeloablative regimen. Etoposide 60 mg/kg was given on Day −3 as an infusion over 5 h. Melphalan 140 mg/m$^2$ was infused over 30 min on Day −2. Reinfusion of stem cells from bone marrow and/or peripheral collection occurred on Day 0. Engraftment time (absolute neutrophil count over $0.5 \times 10^9$/l) was a median of 16 days (range 12–25 days). Total body irradiation was not used for any of the patients.

*Statistic analysis*

Patients were followed until death or until 15 March 2001. Parametric and nonparametric tests were used to compare results. Survival curves, constructed with the date of ASCT as the starting point, were computed according to the Kaplan–Meier method, and differences in survival were compared with the log-rank test for censored data. The Chi-square test was used to compare median survivals. All $P$ values are two-sided.

### Results

### *Toxicity*

Twenty-four patients received HDCT and ASCT between 1988 and 1998. All patients experienced the usual mild to moderate toxicities of mucositis, nausea, fever and neutropenia. Of these, two patients (8%) died of direct complications from bone marrow transplant. One patient suffered a cardiorespiratory arrest at the time of stem cell infusion. The cause for this was unknown even after autopsy was performed. No residual disease was found at postmortem examination. The patient was 36 years old and had an original diagnosis of spindle cell sarcoma in complete remission following seven cycles of doxorubicin. A second patient died of complications of severe veno-occlusive disease and hepatorenal failure 3 months following transplant. The patient was 20 years old and had an original diagnosis of PNET. He had been heavily pretreated (16 cycles of chemotherapy) and had minimal residual disease at time of ASCT. One patient died of an idiopathic pulmonary embolus 5 months following ASCT. He was 54 years old, had Ewing's sarcoma and had no evaluable disease at the time of autotransplant or autopsy.

### *Survival*

Seventeen of 24 patients have now died of their disease. Of the four patients still alive, three are free of disease following transplant. One patient relapsed in the lungs 8 months following transplant and is still alive with disease 32 months later. Overall, the median survival is 10 months (range, 0–137+ months). Of the 18 patients who relapsed, the median time to relapse was 6 months (range, 2–59 months). Ten of these patients had no evaluable disease at time of ASCT, while the other eight had persistent/progressive disease. There was no difference in time to relapse (TTR) if patients were in CR at time of ASCT when compared to patients not in CR, with a median TTR of 6 months (range, 3–59 months) versus 5 months (range, 2–13 months), respectively. Extent of disease at diagnosis also did not seem to affect TTR post ASCT. The Kaplan–Meier survival curve of all patients who received ASCT is shown in Fig. 1.

For the three patients who are free of recurrent disease, the median follow-up is 87 months (range, 75–137 months). Prior to transplant, all three patients had no evaluable disease. Two patients had Ewing's sarcoma (at first presentation, one had metastatic disease to lungs and bones while the other had locally advanced disease) and the third patient had locally advanced alveolar rhabdomyosarcoma. With respect to age, two of the patients were in their mid-thirties at time of diagnosis and ASCT, while the third patient was 11 years at diagnosis and 13 years at transplant. Age therefore did not appear to affect outcome in this series, either on the basis of efficacy or toxicity. The fourth patient, who is alive with recurrence, had an original diagnosis of Ewing's sarcoma metastatic to the lungs treated with chemotherapy and metastatectomy, and also had no evaluable disease at time of ASCT. Figure 2 shows the Kaplan–Meier survival curves of patients according to their disease status at time of ASCT. The difference between the two curves almost reaches statistical significance, with a two-sided $P$ value of 0.07. The 5-year survival estimates are 30% for patients with no evaluable disease (NED) compared to 0% in the group with evaluable disease. The median survivals for both groups, however, are virtually identical at 10 and 11 months, respectively.

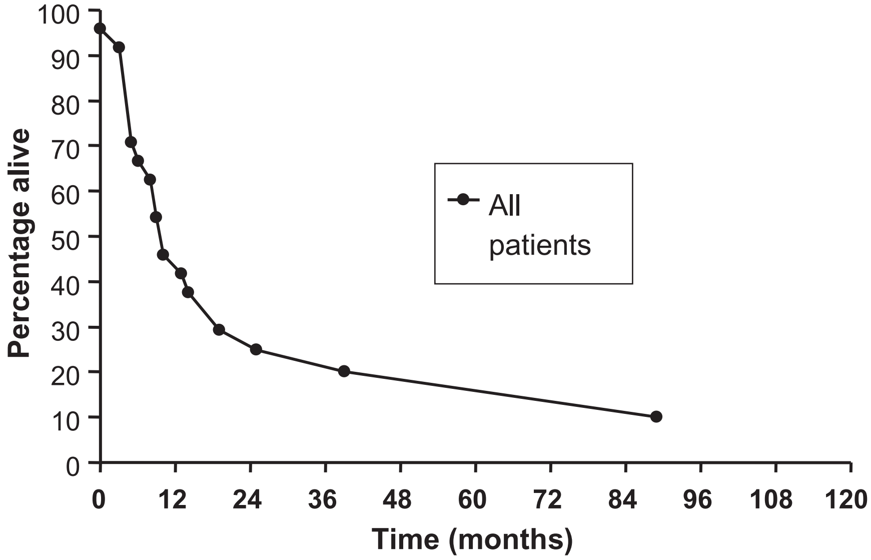

**Fig. 1.** *Kaplan–Meier survival curve of overall survival for all patients. Note that the median survival is 10 months and that no plateau has yet to be discerned.*

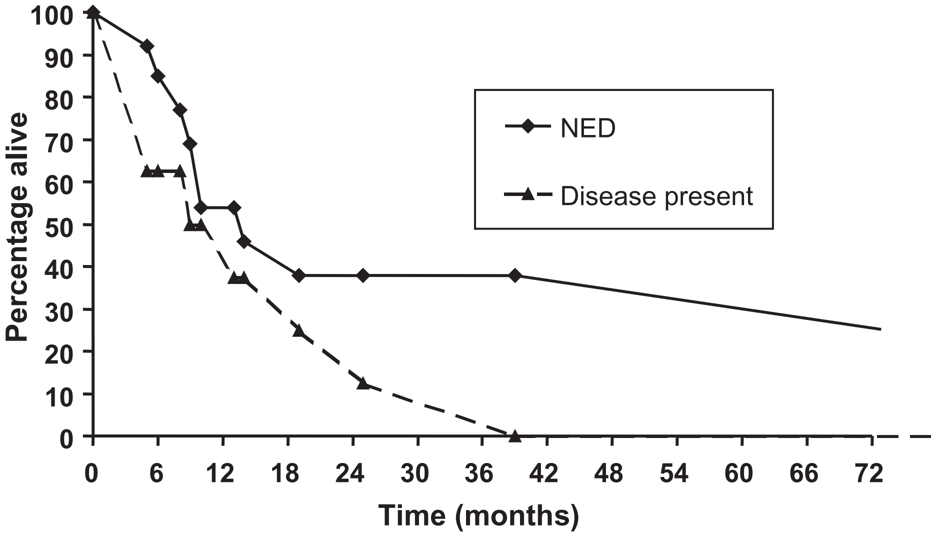

**Fig. 2.** *Kaplan–Meier survival curves of survival according to disease status. NED, no evaluable disease; P = 0.07.*

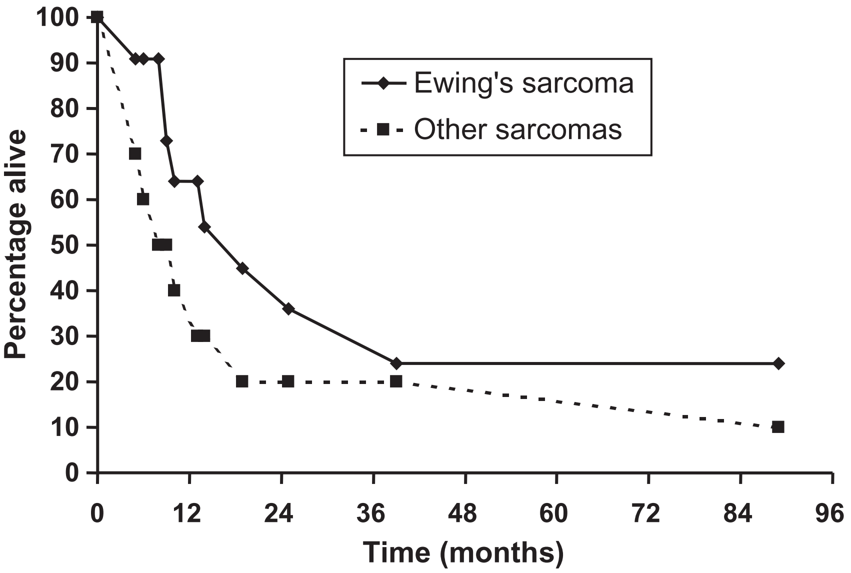

**Fig. 3.** *Kaplan–Meier survival curves according to histological type at time of diagnosis. P = non-significant.*

Figure 3 shows the survival curves according to diagnosis. The median survival for patients with Ewing's sarcoma was 17.5 months compared to 8 months for all other types. However, with small numbers the *P* value is non-significant and the curves are very similar. Furthermore, the 5-year survival rate estimates were 22 and 17%, respectively.

### Discussion

Despite best efforts and aggressive therapy, most patients treated with high-dose melphalan and etoposide in this series have now died of their disease or experienced a recurrence. The overall survival observed here is no better than that found in reports from the published literature. Furthermore, the treatment-related mortality was at least 8%. This is higher than the 1–2% transplant-related mortality observed in most series. A possible reason for this may be the small sample size of our series. However, one of the two patients had been heavily pretreated which might explain his excessive morbidity. It is worth noting that no patients died of infection, the most common cause of death post transplant. In our series, specific histological subtypes like Ewing's sarcomas did not appear to fare better. However, this remains unclear in view of our small numbers. Age also did not appear to be a major predictor of response to this treatment modality.

There was clearly no benefit from HDCT and ASCT if patients were not in complete remission. This is consistent with findings of most of the previously published series.[19,22,23,27,28,32] The four patients who are still alive had no evaluable disease at time of transplant, and in this situation, HDCT was used as consolidation. It is possible that these patients may have had a similar good outcome independent of consolidative therapy. The patient who relapsed 8 months post-ASCT underwent resection of his recurrent pulmonary metastases, and is still alive 32 months later. Thus surgical intervention could explain his prolonged survival.

Our data are in contrast with several other series suggesting improved outcome for HDCT and ASCT in patients with advanced sarcoma.[20,21,24,25,27,29,31,32] However, many of these contained a large number of children[24,25,27–30,32] and none were randomized. Some series[25,27] restricted eligibility to patients with Ewing's sarcoma who had no evaluable disease at time of transplant. In a recent series of ASTS, Blay *et al.*[22] evaluated ifosfamide, etoposide and cisplatin (VIC) as a myeloablative regimen. Of the 30 patients treated, eight were in CR at time of BMT and had a 5-year overall survival rate of 75%. The remaining 22 patients had a much lower 5-year survival rate of 5% ($P=0.001$). They concluded that the VIC regimen may be beneficial in patients with CR at time of transplant and should be evaluated prospectively.

The overall 5-year survival rate in our series was 17%. This is similar to the percentage of long-term survivors among patients with ASTS who had achieved CR after conventional chemotherapy in different series: two of 11 (18%) in Dana-Farber Cancer Institute studies of mesna, doxorubicin, ifosfamide and dacarbazine (MAID);[33] and 11 of 60 (18%) patients with follow-up of at least 5 years in the large database of studies of doxorubicin-based chemotherapy in ASTS held by the European Organization for Research and Treatment of Cancer Soft Tissue and Bone Sarcoma Group.[2] In series describing Ewing's sarcomas of bone metastatic at diagnosis, results were similar: 30% survival at 3 years in 122 patients treated in an Intergroup Ewing's sarcoma study (IESS);[34] 18% survival at 3 years in 48 patients with Ewing's sarcoma of bone treated in European Intergroup Cooperative Ewing's sarcoma studies (CESS) 81 and 86.[35] In our series, the 5-year survival rate estimate of 30% seen in patients with no evaluable disease at time of BMT is only marginally better than those numbers. Furthermore, in a retrospective analysis of 287 patients randomized to two European Osteosarcoma Intergroup (EOI) trials, Lewis *et al.*[36] found no indication that received dose or dose-intensity of doxorubicin/cisplatin influenced survival. This provides more evidence that high-dose chemotherapy may not be helpful in patients with advanced sarcoma. Finally, it has now become clear that HDCT followed by autologous stem cell transplant does not improve survival in women with metastatic breast cancer,[37] even though this disease site has well-established chemosensitivity.

In conclusion, in this retrospective series, high-dose chemotherapy was of no benefit to patients with advanced sarcoma and should not be used routinely outside of clinical trials. This has been the policy in most Canadian centres since the mid-late 1990s. The median overall survival was no better than that seen in reports from published literature and treatment-related mortality was at least 8%. It is possible that patients with Ewing's sarcoma may benefit from HDCT plus ASCT, if they have no evidence of disease when given this treatment, and future studies should concentrate on this population. The critical test would be a prospective randomized trial, which would only be feasible with multicenter and/or intergroup cooperation and physicians should consider referring appropriate patients to the ongoing international Euro-Ewing-Intergroup EE99 study. Lastly, it is possible that newer and different combinations of chemotherapeutic agents like the VIC regimen may prove more effective than our regimen of melphalan and etoposide. Autotransplant could be considered as consolidation earlier in the patients' treatment course, in order to circumvent chemoresistance. This may be a reasonable approach since currently, most of the sarcoma regimens are quite lengthy with significant toxicity and impairment of quality of life. Unfortunately, the negative outcomes observed in randomized controlled trials of patients with metastatic breast cancer, despite multiple positive small single-arm studies, may very well predict the results of such trials in patients with advanced sarcomas, particularly as the data from many of the single-arm studies are not very encouraging.

### References

1. Landis S, Murray T, Boden S, Wingo P. Cancer statistics. *CA Cancer J Clin* 1999; 49: 8–31.
2. Blay JY, van Glabbeke M, Verweij J, *et al.* Advanced soft-tissue sarcoma: a disease that is potentially curable for a subset of patients treated with chemotherapy. *Eur J Cancer* 2003; 39: 64–9.
3. Bui NB, Demaille MC, Chevreau C, *et al.* qMAID vs MAID+25% in adults with advanced soft tissues sarcoma (STS): first results of a randomized study of the FNCLCC Sarcoma Group. *Proc Am Soc Clin Oncol* 1998; 17: 517a (abstr 1991).
4. Santoro A, Tursz T, Mouridsen H, *et al.* Doxorubicin versus CyVADic versus ifosfamide plus doxorubicin in first-line treatment of advanced soft tissue sarcomas: a randomized study of the European Organization for Research and Treatment of Cancer Soft Tissue and Bone Sarcoma Group. *J Clin Oncol* 1995; 13: 1537–45.
5. Antman KH, Crowley J, Balcerzak SP, *et al.* An intergroup phase III randomized study of doxorubicin and dacarbazine with or without ifosfamide and mesna in advanced soft tissue and bone sarcomas. *J Clin Oncol* 1993; 11: 1276–85.

6. Edmonson JH, Ryan LM, Blum RG, *et al.* Randomized comparison of doxorubicin alone versus ifosfamide plus doxorubicin or mitomycin, doxorubicin, and cisplatin against advanced soft tissue sarcomas. *J Clin Oncol* 1993; 11: 1269–75.
7. Van Glabbeke M, van Oosterom AT, Oosterhuis JW, *et al.* Prognostic factors in advanced soft tissue sarcoma: an analysis of 2,185 patients treated with anthracycline-containing first-line regimens-A European Organization for Research and Treatment of Cancer Soft Tissue and Bone Sarcoma Group study. *J Clin Oncol* 1999; 17: 150–7.
8. O'Bryan RM, Baker LH, Gottlieb JB, *et al.* Dose response evaluation of Adriamycin in human neoplasia. *Cancer* 1977; 39: 140–8.
9. Borden EC, Amato D, Enterline HT, *et al.* Randomized comparison of Adriamycin regimens for treatment of metastatic soft tissue sarcomas. *J Clin Oncol* 1987; 5: 840–50.
10. Schoenfeld D, Rosenbaum C, Horton J, *et al.* A comparison of Adriamycin versus vincristine and Adriamycin and cyclophosphamide for advanced sarcoma. *Cancer* 1982; 50: 2757–62.
11. Nielsen OS, Dombernowsky P, Mouridsen H, *et al.* High-dose epirubicin is not an alternative to standard-dose doxorubicin in the treatment of advanced soft tissue sarcomas. A study of the EORTC soft tissue and bone sarcoma group. *Br J Cancer* 1998; 78: 1634–9.
12. Van Oosterom AT, Mouridsen HT, Nielson OS, *et al.* EORTC Soft Tissue and Bone Sarcoma Group. Results of randomised studies of the EORTC Soft Tissue and Bone Sarcoma Group (STBSG) with two different ifosfamide regimens in first- and second-line chemotherapy in advanced soft tissue sarcoma patients. *Eur J Cancer* 2002; 38: 2397–406.
13. Demetri GD. High-dose ifosfamide in the treatment of sarcomas of soft tissues and bone. *Semin Oncol* 1996; 23 (Suppl 6): 22–6.
14. Le Cesne A, Antoine E, Spielmann M, *et al.* High-dose ifosfamide: Circumvention of resistance to standard-dose ifosfamide in advanced soft tissue sarcomas. *J Clin Oncol* 1995; 13: 1600–8.
15. Elias AD, Eder JP, Shea T, *et al.* High-dose ifosfamide with mesna uroprotection: a phase 1 study. *J Clin Oncol* 1990; 8: 95–103.
16. Antman KH, Ryan L, Elias A, *et al.* Response to ifosfamide and mesna. 124 previously patiens with metastatic or unresectable sarcoma. *J Clin Oncol* 1989; 7: 126–31.
17. Houghton JA, Cook RL, Pamela JL, *et al.* Melphalan: a potential new agent in the treatment of childhood rhabdomyosarcoma. *Cancer Treat Rep* 1985; 69: 91–6.
18. Pinkerton CR, Philip T, Bouffet E, *et al.* Autologous bone marrow transplantation in paediatric solid tumours. *Clin Haematol* 1986; 15: 187–203.
19. Tanaka K, Matsunobu T, Sakamoto A, *et al.* High-dose chemotherapy and autologous peripheral blood stem-cell transfusion after conventional chemotherapy for patients with high-risk Ewing's tumors. *J Orthop Sci* 2002; 7: 477–82.
20. Bertuzzi A, Castagna L, Nozza A, *et al.* High-dose chemotherapy in poor-prognosis adult small round-cell tumors: clinical and molecular results from a prospective study. *J Clin Oncol* 2002; 20: 2181–8.
21. Kushner B, Meyers PA. How effective is dose-intensive/myeloablative therapy against Ewing's sarcoma/primitive neuroectodermal tumor metastatic to bone or bone marrow? The Memorial Sloan-Kettering experience and a literature review. *J Clin Oncol* 2001; 19: 870–80.
22. Blay JY, Bouhour D, Ray-Coquard I, *et al.* High-dose chemotherapy with autologous hematopoietic stem-cell transplantation for advanced soft tissue sarcoma in adults. *J Clin Oncol* 2000; 18: 3643–50.
23. Elias AD. High-dose therapy for adult soft tissue sarcoma: dose response and survival. *Semin Oncol* 1998; 25: 19–23.
24. Boulad F, Kernan NA, LaQuaglia MP, *et al.* High-dose induction chemoradiotherapy followed by autologous bone marrow transplantation as consolidation therapy in rhabdomyosarcoma, extraosseous Ewing's sarcoma and undifferentiated sarcoma. *J Clin Oncol* 1998; 16: 1697–706.
25. Atra A, Whelan JS, Calvagna V, *et al.* High-dose busulphan/melphalan with autologous stem cell rescue in Ewing's sarcoma. *Bone Marrow Transplant* 1997; 20: 843–6.
26. Stewart DA, Gyonyor E, Paterson AHG, *et al.* High-dose melphalan +/− total body irradiation and autologous hematopoietic stem cell rescue for adult patients with Ewing's sarcoma or peripheral neuroectodermal tumor. *Bone Marrow Transplant* 1996; 18: 315–8.
27. Burdach S, Jürgens H, Peters C, *et al.* Myeloablative radiochemotherapy and hematopoietic stem-cell rescue in poor-prognosis Ewing's sarcoma and rhabdomyosarcoma. *J Clin Oncol* 1993; 14: 2653–65.
28. Horowitz ME, Kinsella TJ, Wexler LH, *et al.* Total-body irradiation and autologous bone marrow transplant in the treatment of high-risk Ewing's sarcoma and rhabdomyosarcoma. *J Clin Oncol* 1993; 11: 1911–8.
29. Dumontet C, Biron P, Bouffet E, *et al.* High dose chemotherapy with ABMT in soft tissue sarcomas: a report of 22 cases. *Bone Marrow Transplant* 1992; 10: 405–8.
30. Pinkerton CR. Megatherapy for soft tissue sarcomas. EBMT experience. *Bone Marrow Transplant* 1991; 7: 120–2.
31. Burdach ST, Peters C, Paulussen M, *et al.* Improved relapse free survival in patients with poor prognosis Ewing's sarcoma after consolidation with hyperfractionated total body irradiation and fractionated high dose melphalan followed by high dose etoposide and hematopoietic rescue. *Bone Marrow Transplant* 1991; 7 (Suppl 2(3)): 95.
32. Hartmann O, Oberlin O, Beaujean F, *et al.* Role of high-dose chemotherapy followed by bone marrow autograft in the treatment of metastatic Ewing's sarcoma in children. *Bull Cancer* 1990; 77: 181–9.
33. Antman KH, Elias A. Dana-Farber Cancer Institute studies in advanced sarcoma. *Semin Oncol* 1990; 17 (Suppl 2): 7–15.
34. Cangir A, Vietti TJ, Gehan EA, *et al.* Ewing's sarcoma metastatic at diagnosis: results and comparisons of two Intergroup Ewing's Sarcoma Strudies. *Cancer* 1990; 66: 887–93.
35. Wessalwoski R, Jurgens H, Bodenstein H, *et al.* Treatment results of Ewing's sarcoma metastatic at diagnosis: a retrospective analysis of 48 patients. *Klin Padiatr* 1988; 200: 253–60.
36. Lewis IJ, Weeden S, Machin D, *et al.* Received dose and dose-intensity of chemotherapy and outcome in nonmetastatic extremity osteosarcoma. *J Clin Oncol* 2000; 18: 4028–37.
37. Stadtmauer EA, O'Neill A, Goldstein LJ, *et al.* Conventional-dose chemotherapy compared with high-dose chemotherapy plus autologous hematopoietic stem-cell transplantation for metastastic breast cancer. *New Engl J Med* 2000; 342: 1069–76.
